# Current clinical and therapeutic issues in measles

**DOI:** 10.1186/1471-2334-13-S1-P106

**Published:** 2013-12-16

**Authors:** Narcisa Nicolescu, Alexandru Crisan, Emilia Nicoară, Ruxandra Laza, Virgil Musta, Melania Oana

**Affiliations:** 1 Victor Babeş University of Medicine and Pharmacy, Timişoara, Romania; 2 Dr. Victor Babeş Clinical Hospital of Infectious Diseases and Pneumology, Timişoara, Romania

## Background

Known since antiquity (the etiological agent was discovered much later), measles continues to be a problem of infectious pathology present in Romania and in many other countries.

## Methods

We have studied a total of 503 cases admitted to the Infectious Diseases II Clinic of Infectious Diseases and Pneumology Hospital of Timişoara between 2011-2012.

## Results

Measles still represents a common disease among infants and young children, fact confirmed in our study by the high number of cases: 331 (65%). The most common complications in children were the respiratory and ENT infections. The number of measles cases was higher in 2011 compared to 2012 which coincides with data reported by other countries.

## Conclusion

The absence of vaccination from multiple reasons explained the increased number of measles cases (with significant variations in county level). Eradication of measles has been proposed by many countries, e.g. USA, France for over 10 years without being actually realized. In 1993, the measles virus was more neurotropic (there were many cases of deadly encephalitis) compared to the virus strain from 2011-2012.

